# Antifungal susceptibility profiles for fungal isolates from corneas and contact lenses in the United Kingdom

**DOI:** 10.1038/s41433-023-02719-1

**Published:** 2023-09-08

**Authors:** Stephen Tuft, Neil R. H. Stone, Matthew J. Burton, Elizabeth M. Johnson, Andrew M. Borman

**Affiliations:** 1 Moorfields Eye Hospital, 162 City Road, London, EC1V 2PD UK; 2 UCL Institute of Ophthalmology, 11-43 Bath Street, London, EC1V 9EL UK; 3 Department of Clinical Microbiology, University College London Hospitals NHS Foundation Trust, 250 Euston Road, London, NW1 2PG UK; 4 International Centre for Eye Health, London School of Hygiene & Tropical Medicine, Keppel St, London, WC1E 7HT UK; 5 UK National Mycology Reference Laboratory, UK Health Security Agency South-West, Bristol, and MRC Centre for Medical Mycology, University of Exeter, Exeter, UK

**Keywords:** Corneal diseases, Antimicrobials

## Abstract

**Objective:**

To report the identification and results of susceptibility testing for fungal isolates from the cornea or contact lens care systems.

**Materials and methods:**

In this retrospective epidemiological study, we searched the results of fungal cultures from cornea or contact lens systems referred for identification and susceptibility testing to the United Kingdom National Mycology Reference Laboratory between October 2016 and March 2022. For each fungal isolate, we recorded the genus and species of the fungus and the minimum inhibitory concentration (MIC) to six antifungal agents available to treat corneal infection (amphotericin, econazole, itraconazole, natamycin, posaconazole, and voriconazole).

**Results:**

There were 600 isolates from 585 patients, comprising 374 (62%) from corneal samples and 226 from contact lenses and care systems, of which 414 (69%) isolates were moulds (filamentous fungi) and 186 (31%) were yeasts. The most frequent moulds isolated were *Fusarium spp* (234 isolates, 39%) and *Aspergillus spp* (62, 10%). The most frequent yeasts isolated were *Candida spp* (112, 19%), predominantly *Candida parapsilosis* (65, 11%) and *Candida albicans* (33, 6%), with 35 isolates (6%) of *Meyerozyma guilliermondii*. In vitro susceptibility was greatest for natamycin (347 moulds tested, mode 4 mg/L, range 0.25–64 mg/L; 98 yeasts tested, mode 4 mg/L, range 0.5–32 mg/L), with susceptibility for 94% for moulds and 99% yeasts. Of the 16 isolates interpreted as highly resistant to natamycin (MIC ≥16 mg/L), 13 were *Aspergillus flavus* complex.

**Conclusions:**

In vitro susceptibility supports the use of natamycin for the empiric treatment of fungal keratitis in the UK.

## Introduction

Fungal keratitis is a major cause of preventable corneal blindness worldwide, with more than a million cases estimated to occur each year [1]. There are substantial regional differences in both the prevalence and the spectrum of the isolated fungi [1, 2]. In low- and middle-income countries (LMIC), particularly in tropical regions, the large majority of cases are caused by the moulds (filamentous fungi) *Fusarium spp*, *Aspergillus spp*, and *Curvularia spp*, with yeasts accounting for < 1% of cases. However, yeasts are frequently identified in cases from high-income countries (HIC). This difference is associated with the identified risk factors for infection. In LMICs with tropical or subtropical climates, mould infection predominantly occurs secondary to eye trauma in young male agricultural workers of low socioeconomic status [3], while in HICs in temperate regions, the principal risks are prescription contact lens wear, trauma, and chronic ocular surface disease. Estimates of the incidence of fungal keratitis in Europe vary between 0.3 and 1.5 cases per million population per year [4–6], where the most common isolates are *Fusarium spp* (range 19–61%), *Aspergillus spp* (7–33%) and yeasts (5–57%) [4–8], However, the number of *Fusarium spp* infections reported from European centres continues to rise, and between 33% and 73% of mould infections are now associated with prescription contact lens wear [4, 5, 7–10].

Clinical signs may support a fungal keratitis diagnosis [11]. However, there is overlap with other forms of microbial infection [12], and it is usual to perform investigations to confirm the nature of the pathogen. In vivo confocal microscopy (IVCM) or tissue stain of a corneal scrape may provide a point-of-care diagnosis [13]. Further tests to identify the fungus include culture, polymerase chain reaction (PCR) and sequencing, although results can take several days. The culture may then be used to establish antifungal susceptibility profiles to polyenes, imidazoles, triazoles and fluorinated pyrimidines. Identifying fungal isolates and antifungal susceptibility testing is relevant to guide therapy and potentially monitor the emergence and spread of resistance. The current best practice is to initiate treatment with topical antifungal drops such as natamycin. Alternatives include topical voriconazole, amphotericin B, econazole and itraconazole, or the antiseptic chlorhexidine.

The UK National Mycology Reference Laboratory (MRL), part of the UK Health Security Agency (UKHSA), provides a comprehensive service for the diagnosis and susceptibility testing of pathogenic moulds and yeast, both isolated at the MRL and referred from other laboratories within the UK. Because of the strong association between fungal keratitis and contact lens wear and because it is common practice to submit contact lenses for culture in cases of suspected fungal keratitis, we present the results from both corneal isolates and contact lens care products (e.g., lenses, lens cases, and storage fluid). We describe the spectrum of identified isolates, the minimum inhibitory concentrations (MIC), and the proportion of isolates reported as susceptible according to established systemic breakpoint thresholds.

## Materials and methods

The Research Ethics Committee of Moorfields Eye Hospital confirmed the study was excluded from review as the data was fully anonymised and the patients were not identifiable to the research team. The study adhered to the tenets of the Declaration of Helsinki. We used the MRL laboratory information management system to identify fungal cultures from ocular sites received between October 2016 and March 2022. We filtered the data for samples that specified the original culture was grown from the cornea, a contact lens, or the contact lens care system (contact lens case or contact lens cleaning or storage fluid). We combined the data for contact lenses and the contact lens care system, with the origin defined henceforth as contact lens. Details of the lens parameters (rigid gas permeable, flexible, etc.) were not available. Corneal samples were considered separately. We excluded duplicate samples, defined as the same organism grown more than once from the same individual, and data from samples that could not be re-cultured by the MRL laboratory. If more than one fungus was cultured from one referred sample, we considered these as separate isolates. If the same fungus was isolated from the cornea and contact lens, this was reported as a corneal isolate. We did not analyse any bacteria or acanthamoeba isolates grown from polymicrobial infections.

We recorded the genus of positive cultures, with the species if possible. Identification of isolates was performed by phenotypic examination, usually in combination with MALDI-TOF MS analyses and/or rDNA sequencing as described previously [14, 15]. The PCR approaches employed during this period have been described [16]. Namely, pan fungal PCR was performed using previously described primers that target the D1/D2 regions of the 28 S large ribosomal subunit, the ITS1 region, and additional loci (actin, RNA polymerase second largest subunit and translation elongation factor 1 where necessary) [14, 15]. Antifungal susceptibility testing was performed using the Clinical & Laboratory Standards Institute (CLSI) broth microdilution method M27-S4 and M38-A2 [17, 18], as described previously [19]. The MIC (mg/L) was used to determine whether an isolate was susceptible, susceptible at increased exposure, or resistant to a range of antifungal agents. The referring physician’s request largely determined the range of antifungal agents tested with each isolate, with an adjustment to be appropriate for the type or site of infection. As a result, the agents included for evaluation varied between isolates, and we did not test all isolates for all antifungal agents.

As antifungal susceptibility profiles were determined using CLSI broth microdilution methodology, MIC values were interpreted using species-specific CLSI breakpoints where available. In the absence of specific breakpoints, MICs were loosely interpreted against those established for *Aspergillus fumigatus* or *Candida albicans*, for moulds and yeasts, respectively, or using normal ranges/epidemiologic cut-off values (ECVs) in the absence of any breakpoints. For moulds, these are: amphotericin B, itraconazole, voriconazole ≤1.0 mg/L susceptible and ≥2.0 mg/L resistant; posaconazole ≤0.125 mg/L susceptible and ≥0.25 mg/L resistant; econazole ≤2 and 4 mg/L susceptible-increased dose (intermediate) and ≥8.0 mg/L resistant; and for natamycin ≤4 mg/L susceptible-increased dose (intermediate) and ≥8.0 mg/L resistant. However, for topical application for fungal keratitis higher breakpoints may be applicable, as the concentration of the antifungal agent in the tear film is likely to exceed the MIC of the antifungal. We evaluated amphotericin B, econazole, itraconazole, natamycin, posaconazole and voriconazole because these agents are commonly used topically to treat fungal keratitis and they are commonly requested by the physician. Results are presented as the mode and range of the MIC for each individual organism-antifungal agent combination. We also recorded the interpretation of susceptibility testing as either susceptible (S), intermediate (I), or resistant (R), but we then amalgamated the results for S and I (susceptible and susceptible at increased dosage). We report the types of yeast and moulds, the MICs, and the susceptibility for both corneal samples and contact lens products, considered separately and in combination. To identify any inter-genus variability, we then performed a further analysis of the MIC and susceptibility of the three most frequently identified types of mould (*Fusarium spp*, *Aspergillus spp*, *Alternaria spp*) and the three most identified yeasts (*Candida albicans*, *Candida parapsilosis*, and *Meyerozyma guilliermondii* (*Candida guilliermondii*)).

## Results

There were 706 ocular fungal samples received for identification and susceptibility testing in the 5.5 years under review, referred from over 80 contributing centres. From these, we excluded 72 isolates where clinical details were consistent with ocular infection, but the sample site was not explicitly specified, 12 samples from vitreous biopsy, and 22 from the conjunctiva. In 15 samples, two different fungi were grown. For the final analysis there were 600 isolates (585 patients), comprising 374 from corneal samples and 226 from contact lenses and lens care systems. There were 414 (69%) isolates of moulds and 186 (31%) isolates of yeasts (supplementary tables 1, 2). For isolates from corneal samples 273 (73%) were mould and 101 (27%) were yeast. For isolates from contact lenses 141 (62%) were mould and 85 (38%) were yeast. Overall, the most frequently isolated genus of mould were *Fusarium spp* (234 of 600 isolates, 39%), followed by *Aspergillus spp* (62, 10%), *Alternaria spp* (12, 2%) and *Purpureocillium spp* (11, 2%) (supplementary table 1). The most frequent isolated genus of yeast were *Candida spp* (112 of 600 isolates, 19%), predominantly *Candida parapsilosis* (65, 11%) and *Candida albicans* (33, 6%), *Meyerozyma guilliermondii (Candida guilliermondii)* (35, 6%) and *Yarrowia lipolytica* (6, 1%) (supplementary table 2). There were differences in the spectrum of isolates from corneal scrapes compared to contact lenses. For the moulds, *Fusarium spp* were less frequently isolated from corneal samples (136 of 374 isolates, 36%) than contact lenses (98 of 226 isolates, 43%), whereas *Aspergillus spp* accounted for 53 (14%) of isolates from cornea but only 9 (4%) of isolates from contact lenses. Of the yeasts, *Candida albicans* was more frequently isolated from cornea (30 of 374 isolates, 8%) compared to contact lenses (3 of 226 isolates, 1%). Conversely, *Meyerozyma guilliermondii (Candida guilliermondii)* was less commonly isolated from corneal samples (7 of 374 isolates, 2%) than contact lenses (28 of 226 isolates, 12%). There was a trend for a reduction in the number of isolates reported each year during the study, particularly moulds. In 2017 there were 217 isolates from cornea and contact lenses, of which 158 (72.8%) were moulds, while in 2021 there were 55 isolates, of which 30 (54.5%) were moulds (supplementary table 3A).

The combined results for MIC and susceptibility testing for all isolates from cornea and contact lenses tested against six antifungals are presented in Table 1. There was not a marked difference between any of the MIC or susceptibility values for isolates from contact lenses compared to cornea. The broadest spectrum of activity, estimated as the proportion of isolates reported as susceptible, was achieved by natamycin (95.1%), followed by voriconazole (80.8%). Because there is uncertainty about the relevance of systemic breakpoints to topically applied antifungals, we looked for the types of isolates with an MIC ≥16 mg/L for each antifungal, an extremely elevated MIC that would likely predict clinical failure. For natamycin, 13 (81%) of the 16 highly resistant isolates were *Aspergillus flavus* complex and one was *Aspergillus tamnarii*, while none were *Fusarium spp*. One isolate of *Candida albicans* was resistant to natamycin (MIC 32 mg/L), but it was susceptible to amphotericin and voriconazole. In contrast, for voriconazole, 6 (75%) of the 8 highly resistant isolates were *Fusarium spp*, and two were *Lomentospora prolificans*, but none were *Aspergillus spp*. Seven of eight tested isolates of *Purpureocillium lilacinum* were also considered to be resistant to natamycin at a lower MIC of 8 mg/L, and one corneal isolate of a terverticillate *Penicillium* had an MIC of ≥16 mg/L to both natamycin and voriconazole (supplementary table 4).

**Table 1:** Results of in vitro measurement of the minimum inhibitory concentration (mg/L) and the reported susceptibility of ocular fungal isolates against selected antifungal agents frequently used to treat corneal infection.

		Amphotericin	Econazole	Itraconazole	Natamycin	Posaconazole	Voriconazole
**Cornea and contact lens combined**							
**All isolates**	MIC [*N*, mode, (range)]	509, 1, (< 0.03–> 16)	280, 2; > 64, (< 0.125– > 64)*	153, 0.25; > 16, (< 0.03–>16)*	445, 4, (0.25–64)	95, 0.125; > 16, (< 0.03–> 16)*	581, < 0.03; 8, (< 0.03–> 16)*
	Susceptibility [*N* (susceptible %)]	515 (77.9)	333 (66.7)	314 (34.7)	451 (95.1)	100 (48.0)	510 (80.8)
**Mould**	MIC [N, mode, (range)]	357, 1, (< 0.03–> 16)	182, 2, (0.125–> 64)	131, 0.25; > 16, (< 0.03–> 16)*	347, 4, (0.25–64)	79, 0.06; > 16, (< 0.03–> 16)*	399, 4, (< 0.03–> 16)
	Susceptibility [*N* (susceptible %)]	363 (69.7)	235 (53.2)	292 (31.1)	353 (94.1)	86 (45.3)	361 (73.4)
**Yeast**	MIC [*N*, mode, (range)]	152, 0.5; > 16, (0.125–> 16)*	97, 1, (< 0.125–8)	22, 0.25, (< 0.03–1)	98, 4, (0.5–32)	16, < 0.03, (< 0.03–0.5)	182, < 0.03; > 16, (< 0.03–16)*
	Susceptibility [*N* (susceptible %)]	152 (97.4)	98 (99.0)	22 (95.5)	98 (99.0)	14 (64.3)	149 (98.7)
**Cornea samples alone**							
**All isolates**	MIC [*N*, mode, (range)]	325, 1, (< 0.03–16)	169, 1, (< 0.125–> 64)	112, 0.25, (< 0.03–> 16)	299, 4, (0.25–64)	53, 0.125 ( < 0.03–> 16)	324, < 0.03, (< 0.03–> 16)
	Susceptibility [*N* (susceptible %)]	330 (78.5)	199 (67.8)	203 (40.4)	305 (93.1)	73 (53.4)	326 (78.8)
**Mould**	MIC [*N*, mode, (range)]	245, 1, (< 0.03–16)	122, 2, (0.25–> 64)	103, 0.25; > 16, (< 0.03–> 16)*	237, 4, (0.25–64)	47, 0.125;0.25, (< 0.03–> 16)*	246, 0.5;4, (< 0.03–> 16)*
	Susceptibility [*N* (susceptible %)]	250 (72.8)	152 (57.9)	194 (37.6)	243 (91.8)	67 (50.7)	248 (73.0)
**Yeast**	MIC [*N*, mode, (range)]	80, 0.5, (0.25–4)	47, 1, (< 0.125–4)	9, < 0.03, (< 0.03–0.5)	62, 4, (0.5–32)	6, 0.125, (< 0.03–0.5)	78, < 0.03, (< 0.03–1)
	Susceptibility [*N* (susceptible %)]	80 (96.3)	47 (100)	9 (100)	62 (98.4)	6 (83.3)	78 (97.4)

*N* of isolates number tested, % percentage, *in these samples the minimum inhibitory concentration was bimodal, with the two values separated by a semicolon.

Values for susceptible and intermediate (susceptible at increased dosage) have been combined.

The results of MIC and susceptibility testing for the three most frequently identified mould and yeast samples tested against six antifungals are shown in Table 2. There was no major difference between the pattern of susceptibility between isolates from cornea compared to contact lenses (data not shown). The results again show the relatively poor activity of natamycin against *Aspergillus spp* (74% susceptible) compared to voriconazole (100%) and amphotericin (96%). Against *Aspergillus spp* natamycin had the lowest proportion susceptible of any of the six antifungals reported. For *Fusarium spp* all samples tested (100%) were susceptible to natamycin compared to 60% for voriconazole and 67% for amphotericin. All isolates of *Candida parapsilosis* and *Meyerozyma guilliermondii* (*Candida guilliermondii*) were susceptible to natamycin, voriconazole, and amphotericin. The complete data for MIC and susceptibility for all groups of isolates tested against the six antifungals is presented in Supplementary table 4. Susceptibility results for each of the five calendar years where there was complete data is presented in Supplementary table 3B.

**Table 2:** Results of the minimum inhibitory concentration and reported susceptibility of six most common groups of mould and yeast isolates against antifungal agents frequently used to treat corneal infection.

	Amphotericin	Econazole	Itraconazole	Natamycin	Posaconazole	Voriconazole
**Aspergillus spp**						
MIC [N, mode, (range)]	58, 0.25;0.5, (0.06–2)*	29, 1, (0.25–32)	45, 0.25, (0.06–> 16)	54, 4, (1–64)	17, 0.06, (0.06–0.25)	58, 0.5, (0.25–2)
Susceptibility [N (susceptible %)]	58 (96.6)	29 (93.1)	45 (95.6)	54 (74.1)	17 (100)	58 (100)
**Fusarium spp**						
MIC [N, mode, (range)]	212, 1, (0.06–4)	107, 4, (0.25–> 64)	33, > 16, (0.5–> 16)	210, 4, (0.5–16)	26, > 16, (1–> 16)	206, 4, (0.05–32)
Susceptibility [N (susceptible %)]	213 (67.1)	152 (38.8)	180 (0.6)	210 (100)	49 (8.2)	211 (59.7)
**Alternaria spp**						
MIC [N, mode, (range)]	10,0.5, (0.03–1)	3, 1, (1– > 64)	7, 0.5, (0.125–1)	10, 2, (0.5–4)	3, 0.25, (0.06–0.25)	10, 8, (0.25–16)
Susceptibility [N (susceptible %)]	10 (100)	4 (50)	7 (100)	10 (100)	3 (100)	10 (50)
**Candida albicans**						
MIC [N, mode, (range)]	26, 0.5, (0.25–1)	13, < 0.125, (< 0.125–0.25)	2, < 0.03, (< 0.03)	22, 4, (2–32)	1, < 0.03, (< 0.03)	24, < 0.03, (< 0.03)
Susceptibility [N (susceptible %)]	26 (100)	13 (100)	2 (100)	22 (95.5)	1 (100)	24 (100)
**Candida parapsilosis**						
MIC [N, mode, (range)]	54, 0.5, (0.25–1)	36, 1, (< 0.125–4)	8, < 0.03, (0.03–0.06)	32, 4, (4–16)	4, < 0.03, (< 0.03–0.125)	56, < 0.03, (< 0.03–0.06)
Susceptibility [N (susceptible %)]	54 (100)	36 (100)	8 (100)	32 (100)	4 (100)	56 (100)
**Meyerozyma guilliermondii**						
MIC [N, mode, (range)]	29, 0.25, (0.125–1)	19, 2, (0.25–8)	5, 0.25, (0.25–1)	15, 4, (1–4)	5, 0.125, (0.06–0.5)	28, 0.125, (< 0.03–0.5)
Susceptibility [N (susceptible %)]	29 (100)	19 (94.7)	5 (80.0)	15 (100)	5 (80)	28 (100)

N of isolates number tested, *in this sample the minimum inhibitory concentration was bimodal, with values separated with a semicolon.

Values for susceptible and intermediate (susceptible at increased dosage) have been combined.

## Discussion

In this large retrospective case series, we report the identification and susceptibility of isolates referred to the UK National Mycology Reference Laboratory. Because contact lens wear is a major risk for fungal keratitis in the UK and Europe [4, 5, 9, 10], we have analysed isolates grown from contact lenses as well as those grown from the cornea. Our results show that moulds, especially *Fusarium spp* and *Aspergillus spp*, are the most frequent isolates, but with a substantial proportion (31%) of yeasts. This data confirms the continued importance of yeasts as a cause for fungal keratitis in the UK [6, 9]. There were some differences in the types of isolates from the cornea compared to contact lenses, with *Fusarium spp* more frequently isolated from contact lenses than from the cornea, whereas *Aspergillus spp* were more frequently isolated from the cornea. The predominance of *Fusarium spp* may reflect the ability of this pathogen to survive in soil, on plants, and in aqueous environments. In common with other filamentous fungi, it can contaminate contact lenses, survive in biofilms, and penetrate soft contact lens materials [20]. Although *Fusarium spp* have been associated with epidemics of infection related to soft contact lens care solutions [21, 22], the genetic diversity of the isolates suggested an environmental source for the fungi [21]. We also identified *Meyerozyma guilliermondii* (*Candida guilliermondii)* more frequently from contact lenses than corneas, although we are not aware that contact lenses are a reported risk for infection by this pathogen. In contrast to series from LMICs [23], *Curvularia spp* was an uncommon isolate.

We have also reported the susceptibility of these isolates to the antifungal agents that are commonly available to treat fungal keratitis in the UK. Of these, only natamycin 5% (50 mg/L) is licensed, while the other agents are available as compounded products from manufacturing pharmacies. Because there were no major differences in susceptibility between isolates from the cornea or contact lenses, we combined the results of the two groups to give added power for further analysis. We confirm that natamycin has the highest proportion of mould and yeast reported as susceptible (95.1%) followed by voriconazole (80.8%) (Table 1). For moulds, natamycin susceptibility was superior to voriconazole (94.1% vs 73.4%), with *Aspergillus flavus* accounting for almost all the moulds resistant to natamycin. For *Fusarium spp*, all 210 isolates tested were susceptible to natamycin, compared to 59.7% that were susceptible to voriconazole. In contrast, for the *Aspergillus spp*, voriconazole was more effective than natamycin (susceptibility 100% vs 74.1%). For yeast isolates, the proportions of isolates susceptible to amphotericin, econazole, itraconazole, natamycin and voriconazole were similar (range 97.4–99.0%). One isolate of *Candida albicans* was resistant to natamycin (MIC 32 mg/L). Amphotericin B (77.9% overall susceptibility) was less effective against moulds (69.7% susceptible) but an effective treatment for most yeast (96.4% susceptible). Although only a minority of isolates were tested against posaconazole, the proportion of susceptible yeasts (64.3%) was the lowest of the antifungals.

Recommendations for treating fungal keratitis in the UK have, in part, been developed from well-conducted RCTs performed in India and Nepal, which predominantly recruited cases with mould infections and only a tiny minority with a yeast isolate. These show evidence that natamycin is more effective than voriconazole [24, 25], with a difference due to the superior effect of natamycin on *Fusarium spp* [25]. Importantly, with treatment with natamycin 5%, an infection with *Aspergillus spp* had been identified as a predictor for a poor outcome [26], with a recommendation that future RCTs should evaluate the treatment effect of the antifungal on different fungal species [27]. There is also evidence that natamycin 5% is superior to itraconazole 1% [28], but with no evidence of a difference between natamycin and econazole [29]. There are no RCTs comparing amphotericin B with another topical antifungal agent. Evidence from clinical trials is supported by in vitro susceptibility testing, which confirms that some antifungals perform significantly better against particular organisms, such that the MIC of a fungal isolate is a likely predictor of the clinical response; e.g., in vitro susceptibility is associated with an improved outcome for natamycin but not voriconazole [30, 31]. For the most common types of mould infections, *Fusarium spp* isolates are more susceptible in vitro to natamycin than voriconazole, while *Aspergillus spp* are less susceptible to natamycin [30, 32–34]. There may even be differences within a genus, as *Fusarium solani* is reported to have a relatively high MIC for voriconazole (16 mg/L) compared to non-solani species (4 mg/L) [10, 35–38], with an associated delay in healing and a higher rate of complications [39]. MIC data also indicates that itraconazole is likely to be ineffective against *Fusarium spp* [33]. While the MIC for amphotericin against *Fusarium spp* is lower than for either voriconazole or natamycin, the proportion that are classified as resistant is higher, emphasising that a direct comparison of MICs between antifungals is invalid due to differences in achievable tissue concentrations and clinical breakpoints [38]. The pharmacokinetics and pharmacodynamics of an antifungal helps determine the clinical response. Evidence in rabbits indicates that there is poor penetration of topical amphotericin through an intact epithelium, although natamycin can do so [40]. Data of corneal penetration by topical antifungals in humans relates almost exclusively to the use of topical voriconazole on a non-inflamed eye with an intact epithelium. These studies have used a range of topical dosing but report a range of mean concentration in the aqueous of between 1.9 and 7.5 mg/L [41–44]. The single study on inflamed eyes with fungal infection reported a voriconazole concentration in the aqueous of between 2.93 and 3.40 mg/L after combined oral and topical treatment [45]. We could find no comparable data for the penetration of topical natamycin in the human eye, but after hourly administration of natamycin 5% drops to the epithelialized rabbit eye the corneal concentration was estimated at approximately 10.6 mg/L [46].

A review of management guidelines for fungal keratitis may be indicated if there is a change in the spectrum of isolated pathogens or an increase in antifungal resistance. In systemic infections, there is a trend toward infection with non-*albicans* species of *Candida* [47], and an increase in the resistance of *Aspergillus fumigatus* to azoles [48], linked to their use in agriculture [49]. In this context the study of ocular isolates is relevant because some fungi that show emerging resistance, such as *Candida glabrata* and *Candida auris*, are currently rare corneal pathogens [47]. Although there are relatively few studies of corneal isolates [32, 39, 50, 51], an increase in resistance for natamycin and voriconazole against *Fusarium spp* and *Aspergillus spp* has been reported [52]. We could find no published evidence for a difference in the susceptibility profile of common corneal isolates from the UK compared to isolates from other regions. In the short term, of potentially greater relevance than acquired resistance, has been the marked rise in the proportion of *Fusarium spp* isolates since 2000, a fungus that has relative intrinsic resistance to antifungals. Although it was not the purpose of this study to document changes in risk factors for infection or antifungal susceptibility over time, the dataset should act as a benchmark to monitor future trends.

A strength of this study is the inclusion of samples from numerous centres across the UK, with identification and susceptibility testing using standardised protocols. However, there are limitations. Firstly, there was limited information on associated risk factors for fungal keratitis, such as trauma or chronic ocular surface disease, with no information on whether contact lenses were used for cosmesis or for therapy for ocular surface disease. Secondly, as this is data from a reference laboratory, there is no information on clinical outcomes, which would require linking the results back to the referral centres. We do not know whether all isolates from fungal keratitis in the UK were referred to the MRL, or if there are regional differences in the referral rates, although it is likely most moulds were referred for analysis as they are more difficult to identify and treat. Therefore, this is not an accurate estimate of the incidence of fungal keratitis in the UK as we also do not know the number of additional cases in which there was a high index of suspicion of fungal keratitis supported by IVCM, a positive PCR, or histology, but in which cultures were negative. Interestingly, there was a reduction in the number of isolates, particularly moulds, referred to the MRL during the study period. An effect secondary to the COVID lockdown in the UK may have contributed to this. Also, partly due to requests from the referring clinicians, we did not test all isolates to all the relevant antifungal agents. Finally, although antifungal monotherapy is usual, we have not evaluated the effect of combined antifungal agents for synergy nor looked at susceptibility to chlorhexidine. Concerning the clinical relevance of these results, topically applied antifungals can achieve transiently high concentrations in the tear film, but there is uncertainty about the use of systemic breakpoints or ecological cut-off (ECOFF) to determine susceptibility. More information on the corneal penetration of topically applied natamycin would be helpful.

In conclusion, without relevant local data from RCTs, data on MIC and susceptibility may help refine therapeutic guidelines in the UK. The profile of the isolates and the susceptibility data confirms that natamycin is likely the best option for the empiric treatment of suspected fungal keratitis. However, there is a relatively poor susceptibility for natamycin against isolates of *Aspergillus flavus*. Adopting an overall MIC of ≥16 mg/L as representing an isolate extremely unlikely to respond to natamycin, 16 of 445 (3.6%) of cases tested in this series would have received an ineffective treatment until identification and susceptibility results were available. We therefore support the suggestion that future randomized controlled trials of treatment outcomes should be analysed according to mould genus. If it were possible to distinguish *Aspergillus spp* from *Fusarium spp* at the point of first patient contact, using deep learning from images from IVCM [53] or DNA sequencing, modifying therapy early by the early addition or substitution of voriconazole for natamycin would be an option.

## Summary

### What was known before

- Fungal keratitis is a major cause of preventable corneal blindness worldwide Clinical guidelines have been developed from clinical trials in Low- and Middle Income countries However, these studies mainly concern filamentous fungus infection with few cases of yeast infection. Yeast is a common corneal pathogen in the UK.

### What this study adds

- We present data on the types of fungi isolated from cornea and contact lenses in the UK, with the minimum inhibitory concentration and susceptibility to six commonly used antifungals The data supports the use of natamycin as the first line treatment for suspected fungal keratitis Consider changing treatment to voriconazole if an Aspergillus spp is identified.

### Supplementary information

Supplementary Table 1 R1

Supplementary Table 2 R1

Supplementary Table 3 R1

Supplementary Table 4 R1

## Data Availability

All data generated or analysed during this study are included in this published article and its supplementary information files.
