# Supplementary material for: Antifungal susceptibility profiles for fungal isolates from corneas and contact lenses in the United Kingdom
Source: Eye (Lond). 2023 Sep 8;38(3):529–36. doi: 10.1038/s41433-023-02719-1 (PMC10858215; doi:10.1038/s41433-023-02719-1)
Supplement: Supplementary file 1 — Supplementary Table 1 R1 [file 41433_2023_2719_MOESM1_ESM.pdf]

Supplementary Table 1
Identification of the 414 isolates of mould (filamentous fungus) grown from corneal and contact lens samples referred for identification and susceptibility testing between October 2016 and March 2022. Results for contact lenses include isolates from lens cases and lens care solutions. Percentage figures in brackets are the percentage of all 600 isolates. N number.

Mould identification	CONTACT LENS (N)	%	CORNEA (N)	%	Grand Total (N)	%
Acremonium spp	3	(0.5)	6	(1.0)	9	(1.5)
Acrophialophora spp			1	(0.2)	1	(0.2)
Alternaria spp	3	(0.5)	9	(1.5)	12	(2.0)
Arthrinium phaeospermum			1	(0.2)	1	(0.2)
Arthrographis kalrae			1	(0.2)	1	(0.2)
Aspergillus clavatus	1	(0.2)			1	(0.2)
Aspergillus flavus complex	2	(0.3)	14	(2.3)	16	(2.7)
Aspergillus fumigatus complex	4	(0.7)	30	(5.0)	34	(5.7)
Aspergillus nidulans complex			1	(0.2)	1	(0.2)
Aspergillus niger complex	1	(0.2)	5	(0.8)	6	(1.0)
Aspergillus tamarii			1	(0.2)	1	(0.2)
Aspergillus versicolor complex	1	(0.2)	2	(0.3)	3	(0.5)
Aureobasidium pullulans			1	(0.2)	1	(0.2)
Basidiomycete	1	(0.2)	3	(0.5)	4	(0.7)
Beauveria spp			2	(0.3)	2	(0.3)
Bipolaris hawaiiensis			1	(0.2)	1	(0.2)
Bipolaris spp	1	(0.2)			1	(0.2)
Bjerkandera adusta			2	(0.3)	2	(0.3)
Cephalosporium spp	1	(0.2)			1	(0.2)
Ceriporia lacerata			1	(0.2)	1	(0.2)
Chaetomium spp			2	(0.3)	2	(0.3)
Cladophialophora boppii			1	(0.2)	1	(0.2)
Cladosporium sphaerospermum	1	(0.2)			1	(0.2)
Cladosporium spp	2	(0.3)	1	(0.2)	3	(0.5)
Clonostachys rosea			1	(0.2)	1	(0.2)
Corynascus sepedonium			1	(0.2)	1	(0.2)
Curvularia spp	1	(0.2)	1	(0.2)	2	(0.3)
Exophiala oligosperma	1	(0.2)	1	(0.2)	2	(0.3)
Exophiala spp			1	(0.2)	1	(0.2)
Fusarium dimerum	1	(0.2)			1	(0.2)
Fusarium dimerum complex	4	(0.7)	2	(0.3)	6	(1.0)
Fusarium moniliforme complex	1	(0.2)			1	(0.2)
Fusarium oxysporum complex	8	(1.3)	5	(0.8)	13	(2.2)
Fusarium solani complex	3	(0.5)	8	(1.3)	11	(1.8)

Mould identification	CONTACT LENS (N)	%	CORNEA (N)	%	Grand Total (N)	%
Fusarium spp	81	(13.5)	121	(20.2)	202	(33.7)
Gibberella fujikuroi			1	(0.2)	1	(0.2)
Irpex lacteus			1	(0.2)	1	(0.2)
Lecanicillium lecanii	1	(0.2)			1	(0.2)
Lomentospora prolificans			2	(0.3)	2	(0.3)
Metarrhizium spp			1	(0.2)	1	(0.2)
Neosartorya hiratsukae	1	(0.2)	2	(0.3)	3	(0.5)
Neoscytalidium hyalinum			1	(0.2)	1	(0.2)
Ochroconis tshawytschae			1	(0.2)	1	(0.2)
Penicillium chrysogenum	1	(0.2)	1	(0.2)	2	(0.3)
Penicillium spp	1	(0.2)	3	(0.5)	4	(0.7)
Peniphora lycii			1	(0.2)	1	(0.2)
Phaeoacremonium krajdenii			1	(0.2)	1	(0.2)
Phanerochaete sordida			1	(0.2)	1	(0.2)
Phoma spp	2	(0.3)	6	(1.0)	8	(1.3)
Purpureocillium lilacinum	3	(0.5)	7	(1.2)	10	(1.7)
Purpureocillium spp	1	(0.2)			1	(0.2)
Pyrenochaeta unguis-hominis			1	(0.2)	1	(0.2)
Pyrenochaetopsis leptospora			1	(0.2)	1	(0.2)
Rhinocladiella similis	1	(0.2)			1	(0.2)
Sarocladium kiliense	4	(0.7)	2	(0.3)	6	(1.0)
Sarocladium spp	1	(0.2)			1	(0.2)
Sarocladium strictum	1	(0.2)	1	(0.2)	2	(0.3)
Scedosporium apiospermum complex			5	(0.8)	5	(0.8)
Scedosporium de hoogii			2	(0.3)	2	(0.3)
Tilletiopsis spp	1	(0.2)	2	(0.3)	3	(0.5)
Tintelnotia destuctans			1	(0.2)	1	(0.2)
Verticillium spp	1	(0.2)			1	(0.2)
Unidentified mould	1	(0.2)	3	(0.5)	4	(0.7)
**Grand Total**	**141**	**(23.5)**	**273**	**(45.5)**	**414**	**(69.0)**
