# Supplementary material for: Antifungal susceptibility profiles for fungal isolates from corneas and contact lenses in the United Kingdom
Source: Eye (Lond). 2023 Sep 8;38(3):529–36. doi: 10.1038/s41433-023-02719-1 (PMC10858215; doi:10.1038/s41433-023-02719-1)
Supplement: Supplementary file 2 — Supplementary Table 2 R1 [file 41433_2023_2719_MOESM2_ESM.pdf]

Supplementary Table 2

Identification of the 186 isolates of yeast grown from corneal and contact lens samples referred for identification and susceptibility testing between October 2016 and March 2022. Results for contact lenses include isolates from lens cases and lens care solutions. Previous classification for some *Candida spp* given in brackets. Percentage figures in brackets are the percentage of all 600 isolates. N number.

Yeast species	CONTACT LENS (N)	%	CORNEA (N)	%	Grand Total (N)	%
Candida albicans	3	(0.5)	30	(5.0)	33	(5.5)
Candida dubliniensis			1	(0.2)	1	(0.2)
Candida haemulonii	4	(0.7)	2	(0.3)	6	(1.0)
Candida orthopsilosis	1	(0.2)	3	(0.5)	4	(0.7)
Candida palmioleophila	1	(0.2)			1	(0.2)
Candida parapsilosis	30	(5.0)	35	(5.8)	65	(10.8)
Candida pelliculosa	1	(0.2)			1	(0.2)
Candida tropicalis			1	(0.2)	1	(0.2)
Clavispora lusitaniae (Candida lusitaniae)	1	(0.2)	1	(0.2)	2	(0.3)
Cryptococcus diffluens	1	(0.2)	2	(0.3)	3	(0.5)
Cryptococcus uniguttulatus			1	(0.2)	1	(0.2)
Cutaneotrichosporon curvatus	2	(0.3)			2	(0.3)
Cutaneotrichosporon cutaneum	1	(0.2)			1	(0.2)
Debaryomyces hansenii (Candida famata)	1	(0.2)	2	(0.3)	3	(0.5)
Meyerozyma guilliermondii (Candida guilliermondii)	28	(4.7)	7	(1.2)	35	(5.8)
Naganishia albida			1	(0.2)	1	(0.2)
Naganishia diffluens			3	(0.5)	3	(0.5)
Nakaseomyces glabrata (Candida glabrata)			2	(0.3)	2	(0.3)
Rhodotorula dairenensis	1	(0.2)			1	(0.2)
Rhodotorula mucilaginosa	2	(0.3)	3	(0.5)	5	(0.8)
Saccharomyces cerevisiae			1	(0.2)	1	(0.2)
Trichosporon asahii			1	(0.2)	1	(0.2)
Trichosporon jirovecii	1	(0.2)			1	(0.2)
Trichosporon lactis	1	(0.2)			1	(0.2)
Wickerhamomyces anomalus	2	(0.3)	3	(0.5)	5	(0.8)
Yarrowia lipolytica (Candida lipolytica)	4	(0.7)	2	(0.3)	6	(1.0)
**Grand Total**	**85**	**(14.2)**	**101**	**(16.8)**	**186**	**(31.0%)**
