# Supplementary material for: Antifungal susceptibility profiles for fungal isolates from corneas and contact lenses in the United Kingdom
Source: Eye (Lond). 2023 Sep 8;38(3):529–36. doi: 10.1038/s41433-023-02719-1 (PMC10858215; doi:10.1038/s41433-023-02719-1)
Supplement: Supplementary file 3 — Supplementary Table 3 R1 [file 41433_2023_2719_MOESM3_ESM.pdf]

Supplementary Table 3

A) The number of isolates from cornea and contact lenses received in the five calendar years with complete data

Year	Mould N (%)	Yeast N (%)	Total isolates
2017	158 (72.8)	59 (27.2)	217
2018	100 (76.3)	31 (23.7)	131
2019	53 (67.1)	26 (32.9)	79
2020	39 (60.9)	25 (39.0)	64
2021	30 (54.5)	25 (45.5)	55

B) Susceptibility of moulds and yeasts to natamycin, voriconazole, and amphotericin for isolates analyzed in the five calendar years with complete data

Natamycin

		2017 N (%)	2018 N (%)	2019 N (%)	2020 N (%)	2021 N (%)
**Mould**	R	7 (5.5)	7 (8.6)	5 (10.2)	1 (2.8)	0 (0)
	S+I	121	74	44	35	29
**Yeast**	R	0 (0)	0 (0)	0 (0)	1 (5.9)	0 (0)
	S+I	21	14	15	17	22
**Total***		149	95	64	54	51

Voriconazole

		2017 N (%)	2018 N (%)	2019 N (%)	2020 N (%)	2021 N (%)
**Mould**	R	35 (27.6)	27 (32.9)	22 (43.1)	6 (11.3)	5 (12.5)
	S+I	92	55	29	33	25
**Yeast**	R	1 (1.9)	0 (0)	0 (0)	0 (0)	1 (6.7)
	S+I	52	25	19	20	15
**Total***		180	107	70	59	46

Amphotericin

		2017 N (%)	2018 N (%)	2019 N (%)	2020 N (%)	2021 N (%)
**Mould**	R	59 (45.4)	17 (21.0)	8 (15.4)	2 (5.3)	6 (20.0)
	S+I	71	64	44	36	24
**Yeast**	R	1 (1.9)	1 (4.5)	1 (5)	0 (0)	0 (0)
	S+I	52	21	19	21	17
**Total***		183	103	72	59	47

*Number of isolates tested, R resistant, S susceptible, I susceptible at increased dosage. Results for S and I are combined.
