# Supplementary material for: Antifungal susceptibility profiles for fungal isolates from corneas and contact lenses in the United Kingdom
Source: Eye (Lond). 2023 Sep 8;38(3):529–36. doi: 10.1038/s41433-023-02719-1 (PMC10858215; doi:10.1038/s41433-023-02719-1)
Supplement: Supplementary file 4 — Supplementary Table 4 R1 [file 41433_2023_2719_MOESM4_ESM.pdf]

Supplementary Table 4

Results for minimum inhibitory concentrations (mg/L) and susceptibility for all isolates. The cell is left blank if no test was performed. N number of isolates tested, % percentage of tested samples that were susceptible, *no single mode value – both values given separated by semicolon (;)

Isolate	**Amphotericin** MIC [N, mode (range)] N, susceptible (%)	**Econazole** MIC [N, mode (range)] N, susceptible (%)	**Itraconazole** MIC [N, mode (range)] N, susceptible (%)	**Natamycin** MIC [N, mode (range)] N, susceptible (%)	**Posaconazole** MIC [N, mode (range)] N, susceptible (%)	**Voriconazole** MIC [N, mode (range)] N, susceptible (%)
Acremonium spp	8, 4 (1-4)<br>8, (13)	3, 2 (2)<br>5, (60)	1, 2 (2)<br>6, (0)	8, 4 (2-8)<br>8, (100)	1, 1 (1)<br>1, (100)	8, 1 (0.25-2)<br>8, (100)
Acrophialophora spp	1, 0.5 (0.5)<br>1, (100)	1, 0.25 (0.25)<br>1, (100)	1, 0.25 (0.25)<br>1, (100)	1, 4 (4)<br>1, (100)		1, 0.06 (0.06)<br>1, (100)
Alternaria spp	10, 0.5 (0.03-1)<br>10, (100)	3, 1 (1->64)<br>4, (50)	7, 0.5 (0.125-1)<br>7, (100)	10, 2 (0.5-4)<br>10, (100)	3, 0.25 (0.06-0.25)<br>3, (100)	10, 8 (0.25-16)<br>10, (50)
Arthrinium phaeospermum		1, 0.25 (0.25)<br>1, (100)		1, 0.25 (0.25)<br>1, (100)		1, 2 (2)<br>1, (100)
Arthrographis kalrae	1, 1 (1)<br>1, (100)		1, 1 (1)<br>1, (100)	1, 2 (2)<br>1, (100)	1, 0.5 (0.5)<br>1, (100)	1, 0.25 (0.25)<br>1, (100)
Aspergillus clavatus	1, 0.125 (0.125)<br>1, (100)	1, 32 (32)<br>1, (0)	1, >16 (>16)<br>1, (0)	1, 4 (4)<br>1, (100)		1, 2 (2)<br>1, (100)
Aspergillus flavus complex	14, 1 (0.25-2)<br>14, (93)	7, 1 (0.5-2)<br>7, (100)	11, 0.125 (0.06-0.5)<br>11, (100)	14, 32 (16-64)<br>14, (7)	7, 0.125 (0.06-0.25)<br>7, (100)	14, 0.5 (0.25-1)<br>14, 100
Aspergillus fumigatus complex	33, 0.25 (0.125-1)<br>33, (100)	16, 2 (1-32)<br>16, (94)	25, 0.25 (0.06-1)<br>25, (96)	30, 4 (1-8)<br>30, (100)	9, 0.06 (0.06-0.25)<br>9, (100)	33, 0.5 (0.25-2)<br>33, (100)
Aspergillus nidulans complex	1, 1 (1)<br>1, (100)	1, 0.5 (0.5)<br>1, (100)	1, 0.25 (0.25)<br>1, (100)	1, 4 (4)<br>1, (100)		1, 0.25 (0.25)<br>1, (100)
Aspergillus niger complex	6, 0.06 (0.06-0.25)<br>6, (100)	1, 1 (1)<br>1, (100)	4, 0.5 (0.5)<br>4, (100)	5, 4 (2-4)<br>5, (100)	1, 0.125 (0.125)<br>1, (100)	6, 0.5,1 (0.5-1)<br>6, (100)

Isolate	**Amphotericin**<br>MIC [N, mode (range)]<br>N, susceptible (%)	**Econazole**<br>MIC [N, mode (range)]<br>N, susceptible (%)	**Itraconazole**<br>MIC [N, mode (range)]<br>N, susceptible (%)	**Natamycin**<br>MIC [N, mode (range)]<br>N, susceptible (%)	**Posaconazole**<br>MIC [N, mode (range)]<br>N, susceptible (%)	**Voriconazole**<br>MIC [N, mode (range)]<br>N, susceptible (%)
Aspergillus tamarii	1, 0.25 (0.25)<br>1, (100)	1, 0.5 (0.5)<br>1, (100)	1, 0.125 (0.125)<br>1, (100)	1, 64 (64)<br>1, (0)		1, 0.25 (0.25)<br>1, (100)
Aspergillus versicolor complex	2, 0.5;2 (0.5-2)*<br>2, (50)	2, 0.25;2 (0.25-2)*<br>2, (100)	2, 0.125;0.5 (0.125-0.5)*<br>2, (100)	2, 2;4, (2-4)*<br>2, (100)		2, 0.25;0.5 (0.25-0.5)*<br>2, (100)
Aureobasidium pullulans	1, 0.5 (0.5)<br>1, (100)	1, 0.25 (0.25)<br>1, (100)	1, 0.125 (0.125)<br>1, (100)	1, 4 (4)<br>1, (100)		1, 0.25 (0.25)<br>1, (100)
Basidiomycete	1, 0.5 (0.5)<br>1, (100)	1, 0.25 (0.25)<br>1, (100)	1, 0.25 (0.25)<br>1, (100)	1, 4 (4)<br>1, (100)		1, 0.25 (0.25)<br>1, (100)
Beauveria spp	2, 2,4 (2-4)<br>2, (0)	1, 0.5 (0.5)<br>1, (100)	1, 0.25 (0.25)<br>2, (50)	2, 4;8 (4-8)*<br>2, (100)	1, 0.5 (0.5)<br>1, (100)	2, 0.25,1 (0.25-1)<br>2, (100)
Bipolaris hawaiiensis	1, 1 (1)<br>1, (100)			1, 2 (2)<br>1, (100)		1, 1 (1)<br>1, (100)
Bjerkandera adusta	2, 0.125 (0.125)<br>2, (100)	1, 0.25 (0.25)<br>1, (100)	2, 0.25;0.5 (0.25-0.5)*<br>2, (100)	2, 1,2 (1-2)<br>2, (100)	1, 2 (2)<br>1, (0)	2, <0.03;0.06 (<0.03-0.06)<br>2, (100)
Candida albicans	26, 0.5 (0.25-1)<br>26, (100)	13, <0.125 (<0.125-0.25)<br>13, (100)	2, <0.03 (<0.03)<br>2, (100)	22, 4 (2-32)<br>22, (95)	1, <0.03 (<0.03)<br>1, (100)	24, <0.03 (<0.03)<br>24, (100)
Candida dubliniensis	1, 0.25 (0.25)<br>1, (100)			1, 8 (8)<br>1, (100)		1, <0.03 (<0.03)<br>1, (100)
Candida haemulonii	4, 1 (0.5->16)<br>4, (75)	3, 0.5 (0.5-2)<br>3, (100)	1, 0.25 (0.25)<br>1, (100)			3, 0.06 (<0.03-0.25)<br>3, (100)
Candida orthopsilosis	3, 0.5 (0.5)<br>3, (100)	3, 2 (0.25-2)<br>3, (100)		4, 4 (4)<br>4, (100)		3, <0.03 (<0.03-0.125)<br>3, (100)
Candida palmioleophila	1, 0.25 (0.25)<br>1, (100)			1, 2 (2)<br>1, (100)		1, 0.125 (0.125)<br>1, (100)

Isolate	**Amphotericin**<br>MIC [N, mode (range)]<br>N, susceptible (%)	**Econazole**<br>MIC [N, mode (range)]<br>N, susceptible (%)	**Itraconazole**<br>MIC [N, mode (range)]<br>N, susceptible (%)	**Natamycin**<br>MIC [N, mode (range)]<br>N, susceptible (%)	**Posaconazole**<br>MIC [N, mode (range)]<br>N, susceptible (%)	**Voriconazole**<br>MIC [N, mode (range)]<br>N, susceptible (%)
Candida parapsilosis	54, 0.5 (0.25-1)<br>54, (100)	36, 1 (<0.125-4)<br>36, (100)	8, <0.03 (<0.03-0.06)<br>8, (100)	32, 4 (4-16)<br>32, (100)	4, <0.03 (<0.03-0.125)<br>4, (100)	56, <0.03 (<0.03-0.06)<br>56, (100)
Candida tropicalis	1, 1 (1)<br>1, (100)	1, 1 (1)<br>1, (100)		1, 2 (2)<br>1, (100)		1, 0.06 (0.06)<br>1, (100)
Cephalosporium spp	1, 1 (1)<br>1, (100)	1, 0.125 (0.125)<br>1, (100)	1, 0.5 (0.5)<br>1, (100)	1, 4 (4)<br>1, (100)	1, 0.06 (0.06)<br>1, (100)	1, 0.125 (0.125)<br>1, (100)
Ceriporia lacerata	1, 0.5 (0.5)<br>1, (100)		1, 0.25 (0.25)<br>1, (100)	1, 2 (2)<br>1, (100)	1, 0.06 (0.06)<br>1, (100)	1, 0.5 (0.5)<br>1, (100)
Chaetomium spp	2, 0.5,1 (0.5-1)<br>2, (100)	2, 0.25,1 (0.25-1)<br>2, (100)	2, 0.5 (0.5)<br>2, (100)	2, 2 (2)<br>2, (100)		2, 0.5 (0.5)<br>2, (100)
Cladophialophora boppii	1, 1 (1)<br>1, (100)			1, 4 (4)<br>1, (100)		1, 0.5 (0.5)<br>1, (100)
Cladosporium sphaerospermum	1, 2 (2)<br>1, (0)	1, 0.5 (0.5)<br>1, (100)	1, 0.25 (0.25)<br>1, (100)	1, 2 (2)<br>1, (100)		1, 0.25 (0.25)<br>1, (100)
Cladosporium spp	1, 1 (1)<br>1, (100)					1, 1 (1)<br>1, (100)
Clavispora lusitaniae (Candida lusitaniae)	2, 0.25;0.5 (0.25-0.5)<br>2, (100)	2, <0.125 (<0.125)<br>2, (100)		1, 4 (4)<br>1, (100)		2, <0.03 (<0.03-1)<br>2, (100)
Clonostachys rosea	1, 1 (1)<br>1, (100)	1, 8 (8)<br>1, (0)	1, 2 (2)<br>1, (0)	1, 4 (4)<br>1, (100)		1, 8 (8)<br>1, (0)
Corynascus sepedonium	1, 0.125 (0.125)<br>1, (100)	1, 1 (1)<br>1, (100)		1, 2 (2)<br>1, (100)		1, 0.25 (0.25)<br>1, (100)
Cryptococcus diffluens	3, 0.5 (0.5)<br>3, (100)	1, 2 (2)<br>1, (100)	1, 0.5 (0.5)<br>1, (100)	1, 4 (4)<br>1, (100)	1, 0.5 (0.5)<br>1, (0)	3, 0.25 (0.125-0.25)<br>3, (100)

**Isolate**	**Amphotericin**<br>MIC [N, mode (range)]<br>N, susceptible (%)	**Econazole**<br>MIC [N, mode (range)]<br>N, susceptible (%)	**Itraconazole**<br>MIC [N, mode (range)]<br>N, susceptible (%)	**Natamycin**<br>MIC [N, mode (range)]<br>N, susceptible (%)	**Posaconazole**<br>MIC [N, mode (range)]<br>N, susceptible (%)	**Voriconazole**<br>MIC [N, mode (range)]<br>N, susceptible (%)
Cryptococcus uniguttulatus	1, 2 (2)<br>1, (0)	1, 4 (4)<br>1, (100)		1, 4 (4)<br>1, (100)		1, 1 (1)<br>1, (100)
Curvularia spp	2, 0.25,0.5 (0.25-0.5)<br>2, (100)	1, 2 (2)<br>1, (100)	2, 0.25 (0.25)<br>2, (100)	2, 1,4 (1-4)<br>2, (100)	1, 0.125 (0.125)<br>1, (100)	2, 0.25,1 (0.25-1)<br>2, (100)
Cutaneotrichosporon curvatus	2, 0.25,0.5 (0.25-0.5)<br>2, (100)	2, 0.5 (0.5)<br>2, (100)		1, 2 (2)<br>1, (100)		2, <0.03;0.125 (<0.03-0.125)*<br>2, (100)
Cutaneotrichosporon cutaneum	1, 0.5 (0.5)<br>1, (100)	1, 1 (1)<br>1, (100)		1, 4 (4)<br>1, (100)		1, 0.06 (0.06)<br>1, (100)
Debaryomyces hansenii (Candida famata)	2, 1 (1)<br>2, (100)	2, 0.25;2 (0.25-2)*<br>2, (100)	1, 0.25 (0.25)<br>1, (100)	2, 0.5,1 (0.5-1)<br>2, (100)		2, <0.03;0.06 (<0.03-0.06)*<br>2, (100)
Exophiala oligosperma	1, 0.5 (0.5)<br>1, (100)	1, 1 (1)<br>1, (100)	1, 0.065 (0.065)<br>1, (100)	1, 2 (2)<br>1, (100)		1, 0.125 (0.125)<br>1, (100)
Fusarium dimerum	1, 1 (1)<br>1, (100)			1, 2 (2)<br>1, (100)	1, >16 (>16)<br>1, (0)	1, 8 (8)<br>1, (0)
Fusarium dimerum complex	7, 1 (0.25-2)<br>7, (86)	2, >64 (>64)<br>4, (0)	3, >16 (>16)<br>6, (0)	7, 2 (2-4)<br>7, (100)	1, >16 (>16)<br>2, (0)	7, 8 (1-16)<br>7, (14)
Fusarium moniliforme complex	1, 1 (1)<br>1, (100)	1, (0)	1, (0)	1, 4 (4)<br>1, (100)		1, (0)
Fusarium oxysporum complex	12, 1 (1-2)<br>13, (62)	8, 2 (2-64)<br>10, (40)	3, >16 (>16)<br>12, (0)	13, 8 (1-8)<br>13, (100)		13, 4 (2-16)<br>13, (92.3)
Fusarium solani complex	11, 1 (0.25-2)<br>11, (82)	5, 4 (2->64)<br>7, (43)	5, >16 (16->16)<br>10, (0)	10, 4 (4-8)<br>10, (100)	2, >16 (>16)<br>3, (0)	10, 4 (2-4)<br>10, (50)

**Isolate**	**Amphotericin**<br>MIC [N, mode (range)]<br>N, susceptible (%)	**Econazole**<br>MIC [N, mode (range)]<br>N, susceptible (%)	**Itraconazole**<br>MIC [N, mode (range)]<br>N, susceptible (%)	**Natamycin**<br>MIC [N, mode (range)]<br>N, susceptible (%)	**Posaconazole**<br>MIC [N, mode (range)]<br>N, susceptible (%)	**Voriconazole**<br>MIC [N, mode (range)]<br>N, susceptible (%)
Fusarium spp	180, 1 (0.06-4)<br>180, (66)	92, 4 (0.25->64)<br>130, (40)	22, >16 (0.5->16)<br>151, (1)	178, 4 (0.5-16)<br>178, (100)	22, >16 (1->16)<br>43, (9)	175, 4 (0.5->16)<br>179, (60.3)
Gibberella fujikuroi	1, 1 (1)<br>1, (100)			1, 4 (4)<br>1, (100)		1, 4 (4)<br>1, (100)
Irpex lacteus	1, 8 (8)<br>1, (0)		1, 0.5 (0.5)<br>1, (100)	1, 1 (1)<br>1, (100)	1, 0.125 (0.125)<br>1, (100)	1, 0.5 (0.5)<br>1, (100)
Lomentospora prolificans	2, 8;>16 (8->16)*<br>2, (0)	2, >64, (>64)<br>2, (0)	2, >16, (>160<br>2, (0)	2, 4 (4)<br>2, (100)		2, 16;>16 ((16->16)*<br>2, (0)
Metarrhizium spp	1, 2 (2)<br>1, (0)	1, 2 (2)<br>1, (100)	1, (0)	1, 8 (8)<br>1, (100)		1, 1 (1)<br>1, (100)
Meyerozyma guilliermondii (Candida guilliermondii)	29, 0.25 (0.125-1)<br>29, (100)	19, 2 (0.25-8)<br>19, (95)	5, 0.25 (0.25-1)<br>5, (80)	15, 4 (1-4)<br>15, (100)	5, 0.125 (0.06-0.5)<br>5, (80)	28, 0.125 (<0.03-0.5)<br>28, (100)
Naganishia diffluens	1, 1 (1)<br>1, (100)	1, 1 (1)<br>1, (100)		1, 2 (2)<br>1, (100)		1, 0.25 (0.25)<br>1, (100)
Nakaseomyces glabrata (candida glabrata)	2, 0.5.1 (0.5-1)<br>2, (100)	1, 0.25 (0.25)<br>1, (100)	1, 0.5 (0.5)<br>1, (100)	2, 4 (4)<br>2, (100)		1, 0.25 (0.25)<br>1, (100)
Neosartorya hiratsukae	3, 0.25 (0.25-0.5)<br>3, (100)	1, 0.5 (0.5)<br>1, (100)	1, 0.125 (0.125)<br>1, (100)	3, 4 (4-8)<br>3, (100)		3, 0.5 (0.5-4)<br>3, (100)
Ochroconis tshawytschae	1, 0.5 (0.5)<br>1, (100)			1, 1 (1)<br>1, (100)	1, 0.125 (0.125)<br>1, (100)	1, 1 (1)<br>1, (100)
Penicillium chrysogenum	2, 0.25;0.5 (0.25-0.5)*<br>2, (100)	2, 0.5 (0.5)<br>2, (100)	2, 0.25;0.5 (0.25-0.25)*<br>2, (100)	2, 2;4 (2-4)*<br>2, (100)		2, 1;2 (1-2)*<br>2, (100)
Penicillium spp	4, 0.25 (0.25-2)<br>4, (75)	1, 1 (1)<br>2, (100)	2, 0.06;0.125 (0.06-0.125)*<br>3, (67)	4, 1 (0.5-32)<br>4, (75)	1, 0.03 (0.03)<br>2, (50)	4, 0.25 (0.25-16)<br>4, (75)

Isolate	**Amphotericin** MIC [N, mode (range)] N, susceptible (%)	**Econazole** MIC [N, mode (range)] N, susceptible (%)	**Itraconazole** MIC [N, mode (range)] N, susceptible (%)	**Natamycin** MIC [N, mode (range)] N, susceptible (%)	**Posaconazole** MIC [N, mode (range)] N, susceptible (%)	**Voriconazole** MIC [N, mode (range)] N, susceptible (%)
Peniphora lycii	1, 0.5 (0.5)<br>1, (100)	1, 0.5 (0.5)<br>1, (100)	1, 0.125 (0.125)<br>1, (100)	1, 2 (2)<br>1, (100)		1, 8 (8)<br>1, (0)
Phaeoacremonium krajdenii	1, 0.25 (0.25)<br>1, (100)			1, 2 (2)<br>1, (100)	1, 0.25 (0.25)<br>1, (100)	1, 0.25 (0.25)<br>1, (100)
Phanerochaete sordida	1, 0.5 (0.5)<br>1, (100)	1, 1 (1)<br>1, (100)	1, 0.5 (0.5)<br>1, (100)	1, 2 (2)<br>1, (100)	1, 0.25 (0.25)<br>1, (100)	1, 0.25 (0.25)<br>1, (100)
Phoma spp	6, 1 (0.03-2)<br>6, (83)	3, 0.5 (0.5)<br>3, (100)	6, 0.25 (0.25-1)<br>6, (100)	6, 2 (0.25-2)<br>6, (100)	3, 0.125 (<0.03-1)<br>3, (100)	5, 0.25 (<0.03-1)<br>5, (100)
Purpureocillium lilacinum	2, 8,16 (8-16)<br>7, (0)	4, 0.5,1 (0.5-1)<br>3, (100)	3, 1 (1-2)<br>4, (50)	1, 8 (8)<br>7, (14)	1, 0.25 (0.25)<br>1, (100)	7, 0.25 (0.25-0.5)<br>7, (100)
Purpureocillium spp	1, 2 (2)<br>1, (0)		1, 0.5 (0.5)<br>1, (100)			1, 0.25 (0.25)<br>1, (100)
Pyrenochaeta unguis-hominis	1, 0.5 (0.5)<br>1, (100)					1, >16 (>16)<br>1, (0)
Rhodotorula mucilaginosa	5, 0.5 (0.25-0.5)<br>5, (100)	4, 4 (0.5-4)<br>4, (100)		2, 4 (4)<br>2, (100)		5, 4 (0.5-16)<br>5, (60)
Saccharomyces cerevisiae				1, 4 (4)<br>1, (100)		
Sarocladium kiliense	6, 2 (2-4)<br>6, (0)	4, >64 (0.25->64)<br>6, (17)	1, >16 (>16)<br>3, (0)	6, 2 (2-4)<br>6, (100)		6, 1 (0.125-2)<br>6, (100)
Sarocladium strictum	2, 1,4 (1-4)<br>2, 50	2, (0)	2, (0)	2, 2;4 (2-4)*<br>2, (100)		2, 1;2 (1-2)*<br>2, (100)
Scedosporium apiospermum complex	5, 1;2 (1-4)*<br>5, (40)	2, 1;2 (1-2)*<br>2, (100)	3, 4 (0.5-4)<br>3, (33)	4, 4 (2-8)<br>4, (100)		5, 0.5 (0.5-1)<br>5, (100)

Isolate	**Amphotericin** MIC [N, mode (range)] N, susceptible (%)	**Econazole** MIC [N, mode (range)] N, susceptible (%)	**Itraconazole** MIC [N, mode (range)] N, susceptible (%)	**Natamycin** MIC [N, mode (range)] N, susceptible (%)	**Posaconazole** MIC [N, mode (range)] N, susceptible (%)	**Voriconazole** MIC [N, mode (range)] N, susceptible (%)
Scedosporium de hoogii	2, 2 (2)<br>2, (0)	1, 0.25 (0.25)<br>1, (100)	2, 0.5 (0.5)<br>2, (100)	2, 2 (2)<br>2, (100)		2, 0.25 (0.25)<br>2, (100)
Tilletiopsis spp	2, 0.125;2 (0.125-2)*<br>2, (50)	1, 2 (2)<br>2, (50)	1, 0.25 (0.25)<br>2, (50)	2, 4 (4)<br>2, (100)		2, 0.06;0.5 (0.06-0.5)*<br>2, (100)
Trichosporon asahii	1, 2 (2)<br>1, (0)					1, <0.03 (<0.03)<br>1, (100)
Trichosporon jirovecii	1, 0.5 (0.5)<br>1, (100)	1, (100)		1, 4 (4)<br>1, (100)		1, <0.03 (<0.03)<br>1, (100)
Trichosporon lactis	1, 0.5 (0.5)<br>1, (100)	1, 0.25 (0.25)<br>1, (100)				1, 0.03 (0.03)<br>1, (100)
Unidentified mould	2, 0.06;0.125 (0.06-0.125)*<br>2, (100)	1, 0.5 (0.5)<br>1, (100)	1, 0.25 (0.25)<br>1, (100)	2, 4 (4)<br>2, (100)		2, 0.125;0.5 (0.125-0.5)*<br>2, (100)
Verticillium spp	1, 2 (2)<br>1, (0)	1, 2 (2)<br>1, (100)	1, >16 (>16)<br>1, (0)	1, 4 (4)<br>1, (100)		1, 2 (2)<br>1, (100)
Wickerhamomyces anomalus	5, 0.25 (0.25)<br>5, (100)	3, 1 (1-2)<br>3, (100)	1, 0.5 (0.5)<br>1, (100)	4, 2,4 (2-4)<br>4, (100)	1, 0.5 (0.5)<br>1, (0)	5, 0.125 (0.125-0.25)<br>5, (100)
Yarrowia lipolytica (Candida lipolytica)	6, 1 (0.5-4)<br>6, (83)	3, 0.5 (0.25-2)<br>3, (100)	2, 0.25;0.5 (0.25-0.5)*<br>2, 100	4, 2 (2-8)<br>4, (100)	2, 0.25 (0.25)<br>2, (0)	6, 0.06 (<0.03-0.125)<br>6, (100)
